# Differential expression and bioinformatics analysis of circRNA in osteosarcoma

**DOI:** 10.1042/BSR20181514

**Published:** 2019-05-17

**Authors:** Yizhe Xi, Mitra Fowdur, Yun Liu, Hao Wu, Maolin He, Jinmin Zhao

**Affiliations:** 1 Division of Spinal Surgery, The First Affiliated Hospital of Guangxi Medical University, Nanning, Guangxi, P.R. China; 2 Guangxi Key Laboratory of Regenerative Medicine, Guangxi Medical University, Nanning, Guangxi, P.R. China; 3 Department of Orthopedic Trauma and Hand Surgery, The First Affiliated Hospital of Guangxi Medical University, Nanning, Guangxi, P.R. China

**Keywords:** bioinformatics, circRNA, osteosarcoma

## Abstract

Aim: This research aims to investigate the expression profile of circRNA in osteosarcoma and to identify the underlying pathogenesis core genes of osteosarcoma.

Methods**:** Illumina HiSeq was used to screen differentially expressed circRNAs between the tumour tissues and paracancerous tissues of three osteosarcoma patients. Bioinformatics analysis was used to analyse their potential functions. Five differentially expressed circRNAs were selected to detect the relative expression level in tumour and paracancerous tissues of ten osteosarcoma patients by real-time PCR. The databases such as DisGeNET and miRWalk were used to collect related genes or miRNAs.

Results: A total of 259 differentially expressed circRNAs were evaluated in patients with osteosarcoma, of which 132 were up-regulated and 127 were down-regulated. Compared with that in paracancerous tissues, circ_32279 and circ_24831 were significantly down-regulated while circ_2137 and circ_20403 were significantly up-regulated in osteosarcoma tissues. The differential expression of circRNA is closely linked to biological processes and molecular functions. The difference in circRNA was mainly linked to the ‘phosphatidylinositol signalling system’ signal pathway and the ‘inositol phosphate metabolism’ signal pathway.

Conclusion: The present study identified a profile of abnormal regulation of circRNA in osteosarcoma. Bioinformatics analysis indicates that the deregulated circRNAs may be related to the occurrence and development of osteosarcoma.

## Introduction

Osteosarcoma is a life-threatening cancer that usually occurs in children and adolescents [1]. Osteosarcoma may occur at the metaphysis of long bones. The most common bones include approximately 40% femur, 20% tibia, 10% humerus and 8% pelvis [2]. Osteosarcoma is developed by the mesenchymal cell line, characterized by producing osteoid tissue and osteoid- or spindle-shaped stromal cells in immature bones produced by tumour cells. The incidence of osteosarcoma in the world is approximately 3–4 per million new cases [3]. At present, the commonly used clinical treatment methods include systemic chemotherapy and local control surgery [4]. With the advancement of surgery and the introduction of neoadjuvant chemotherapy, great improvements have been made in the clinical response and long-range survival rate of osteosarcoma. However, although surgery and neoadjuvant chemotherapy have achieved great progress, most patients still have a poor prognosis. Even after standardized chemotherapy and radical resection of the primary lesions, there is still a high risk of local recurrence or pulmonary metastasis. For patients with metastatic disease or tumour recurrence, the clinical outcomes are even worse [5]. Unfortunately, since there are only a few diagnostic indicators and therapeutic targets in patients with osteosarcoma, the improvement of prognosis is severely limited. Therefore, the identification of effective biomarkers or therapeutic targets in osteosarcoma is a strategy to avoid chemotherapy resistance, reduce lung metastasis and improve clinical prognosis.

CircRNA is a newly recognized class of special noncoding RNA molecules. It mainly consists of endogenous RNA molecules formed by exon transcripts and nonlinear reverse splicing, and circRNA molecules containing introns. Most of the cyclic RNAs are covalently connected with each other by the 3′,5′-phosphodiester bond without a polyadenylated tail. CircRNA has been identified amongst some tumour and cell lines. Accumulating studies show that some circRNAs play an active role in the initiation and development of cancer. Because it is insensitive to nuclease, and thus more stable than linear RNA, circRNA has an obvious advantage in the development and application of new clinical diagnostic markers. It has been reported that more than 1000 kinds of circRNA enrichment were detected in human serum exosomes; circ-KLHDC10 expression levels can be successfully distinguished from colorectal cancer patients and normal individuals [6]. Some circRNAs contain miRNA response elements that act as a competitive endogenous RNA (ceRNA) that binds to miRNAs and act as miRNA sponges in cells, thereby eliminating the miRNA inhibition of target genes and up-regulating the expression levels of target genes. Huang et al. [7] found that silencing circRNA hsa_circ_0000977 inhibits the progression of pancreatic ductal adenocarcinoma by stimulating miR-874-3p and inhibiting PLK1 expression. CircRNA CDR1 competitively binds miR-7, up-regulates Pax6 and MyRIP, and promotes insulin mRNA transcription and the extracellular secretion of protein [8]. CircHIPK3 regulates cell growth through the action of miRNA sponges and miRNAs [9].

Some studies have been published on the correlations between circRNA and osteosarcoma patients. Liu et al. [10] proved that the expression level of Circ-NT5C2 was up-regulated in osteosarcoma tissues and cell lines, and also silently inhibits the growth of tumours in the body. However, the association between significant changes in circRNA expression and the occurrence of osteosarcoma is still unclear [11]. The purpose of the present study is to explore the circRNA expression profile in osteosarcoma, to identify the underlying role of core genes in osteosarcoma pathogeny and to further confirm the relationship between osteosarcoma patients’ circRNA and prognosis.

## Materials and methods

### Clinical specimen acquisition

Specimens of osteosarcoma and paracancerous tissues were obtained from 13 patients undergoing surgery at the First Affiliated Hospital of Guangxi Medical University (Nanning, China). The patients were not treated with radiotherapy or chemotherapy before surgical operation. This research was conducted in line with the 1964 Helsinki Declaration as amended or similar moral criteria. The present study was approved by the ethics committee of the First Affiliated Hospital of Guangxi Medical University, and written informed consent was obtained from the patients. Supplementary Table S1 shows the clinical specimen data of the 13 osteosarcoma patients.

### Preparation of sequencing library and sequencing of circRNA

Three osteosarcoma tissues were randomly sampled out of the 13 specimens, from which total RNA was extracted to test its concentration and purity by Nanodrop 2000. RNA integrity was detected by agarose gel electrophoresis, and the RIN (RNA integrity number) value was determined by Agilent 2100. A single database construction requires a total of 5 μg of RNA, a concentration of ≤200 ng/μl, and an OD260/280 of 1.8 to 2.2. RNase R was utilized to remove linear RNAs. Metal ions were utilized to randomly break the circRNA into small 200 bp fragments. Under the action of reverse transcriptase, a chain cDNA was synthesized by random primers using circRNA as a template. When two chain syntheses were synthesized, the dNTPs reagent used dUTP instead of dTTP to make the base of cDNA second chain containing A/U/C/G. End Repair Mix was added to the flat end, followed by an A base at the 3′ end to connect the Y-shaped joint. CDNA second chains were digested by UNG enzymes to form distinct strand DNA molecules, captured on Illumina flow cytometry and amplified *in situ* as clusters, which were then sequenced for 150 cycles on an Illumina HiSeq Sequencer in accordance with instructions from the manufacturer.

### Sequencing data quality control

The quality of the subsequent analysis is influenced by the sequence of sequencing joint, low quality reading segment, high N rate sequence and short length sequence in the original sequencing data. To ensure the accuracy of subsequent bioinformatics analysis, the raw sequencing data were first filtered to obtain high-quality clean data to ensure that the follow-up analysis was smoothly performed. The quality control process was completed by tools such as SeqPrep (https://github.com/jstjohn/SeqPrep) and Sickle (https://github.com/najoshi/sickle) reference genome alignment.

CircRNA is formed by splicing the donors downstream of exons to splice the receptors upstream of exons. Because the sequence of exons is rearranged, the normal linear ratio cannot obtain the ring-splicing reads. Therefore, it is necessary to compare the reads with a reference according to the linear rule to obtain back-spliced junction reads. The clean read was compared with the human reference genome (hg19, UCSC [12]) with the TopHat2 [13] software. The nonlinear junction reads were accurately analysed by using TopHat-Fusion to compare the nonlinear reads to the reference genome.

### CircRNA identification, quantification and annotation

CIRCexplorer2 [14] was utilized to identify circRNAs, and candidate circRNAs with junction read counts >2 were screened as the final identified circRNAs. Built on the location of the circRNA in the genome and the relationship with the gene, the selected circRNA candidate was annotated with the Ensembl database and RefSeq database, mainly including two parts. The first part was classified according to the circRNA position relationship. The second part was circRNA function annotation, mainly based on the circRNA-hosting gene function annotation.

### Identification of differentially expressed circRNAs

The number of back-spliced reads is used to estimate the amount of circRNA expression. Based on circRNA expression, the quasi-likelihood *F*-test method of the edgeR [15] software package was used for differential expression analysis. Differentially expressed circRNAs between cancer tissue and paracancerous tissue in two paired tissues were screened with defined threshold values >1.0 (|logFC| > 1) and *P* values <0.01 (*P* value < 0.01). The analysis required circRNA to express more than 0 values in at least two samples, and the unqualified circRNA was deleted.

### Utilizing of qRT-PCR

qRT-PCR was performed to verify the accuracy of the circRNA-seq data. According to the manufacturer’s instructions, the PrimeScript RT reagent kit with a gRNA eraser was used to synthesize the cDNA of tumour and normal samples by reverse transcription in the tumour tissue and paracancerous tissue samples of the other ten osteosarcoma patients. qRT-PCR was performed to measure differentially expressed circRNAs on an Applied Biosystems StepOne Plus Real-Time PCR System using SYBR Premix ExTaq. Twelve pairs of randomly selected primers for outward circulating RNA are illustrated in Supplementary Table S2. The qRT-PCR conditions were adjusted as the primary denaturation procedure at 50.0°C for 3 min, 95°C for 10 s, 60°C for 30 s, for 40 cycles, and the last step was slowly heated from 60 to 95°C. The relative expression of every circRNA connected to GAPDH was calculated.

Data are presented as mean ± standard deviation (SD), GraphPad Prism 5.0 (San Diego, U.S.A.) or SPSS 20.0 (Chicago, U.S.A.) was used for all statistical analyses. A significant difference was considered when *P* < 0.05.

### Bioinformatics analysis of differentially expressed circRNAs

The differentially expressed circRNA-hosting gene was analysed by the DAVID tool (V6.8) [16] for its enriched GO function and KEGG pathway analysis. A parameter of enrichment gene count ≥2 and hypergeometric test significance threshold *P* value <0.05 were considered as the result of significant enrichment.

### Interaction between circRNA and miRNA

The miRanda [17] tool was used to perform miRNA-targeting predictions on differentially expressed circRNAs, and the Cytoscape [18] tool was used to construct a network map for targeted miRNAs and circRNAs.

### MiRNA and gene acquisition of osteosarcoma

Related genes or miRNAs were collected through databases such as DisGeNET [19] and miRWalk [20]. For the related genes, the selected data were collected from the following databases: UniProt, CTD gene-disease subsets, PsyGeNET, Orphanet, and HPO-Human Phenotype Ontology. For related miRNAs, osteosarcoma-associated miRNAs were selected in validated disease-miRNA interactions in miRWalk. For the obtained osteosarcoma-related miRNAs and genes, we predicted whether there was a regulatory relationship between them through miRWalk and selected the results shared by miRWalk, miRanda and TargetScan [21] as the final miRNA–gene relationship.

## Results

### Differential expression of circRNA in patients with osteosarcoma

**Figure 1:**
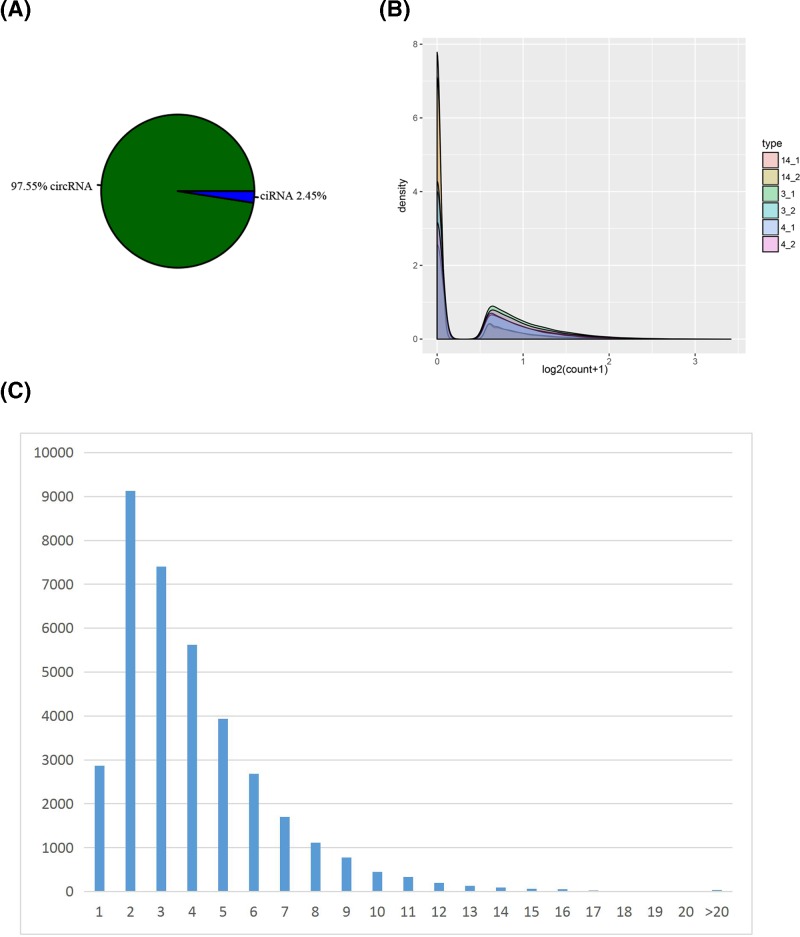
CircRNA expression profile in osteosarcoma tissues (**A**) The source composition of circRNA classified according to the source region. (**B**) CircRNA expression density distribution. (**C**) Distribution of circRNA exons. The horizontal axis indicates the number of exons contained in the circRNA, and the vertical axis indicates the number of circular RNAs corresponding to the number of exons.

CircRNA was sequenced in three osteosarcoma tissues and three paracancerous tissue samples. Through analysis, 36631 circRNAs were identified, corresponding to 8001 genes (hostgene). After annotation, circRNA was divided into intron-derived circular RNA (ciRNA) and exon-derived circular RNA (circRNA) according to its source. Pie charts were utilized to analyse its composition (Figure 1A). The amount of circRNA expression was estimated by the number of back-spliced reads, and the distribution of circRNA expression was shown by a density distribution map (Figure 1B). The peaks of the exon numbers of circRNA are usually located at 2–3 locations, and the following statistical histogram was made according to the number of exons contained in the circRNA (Figure 1C).

**Figure 2:**
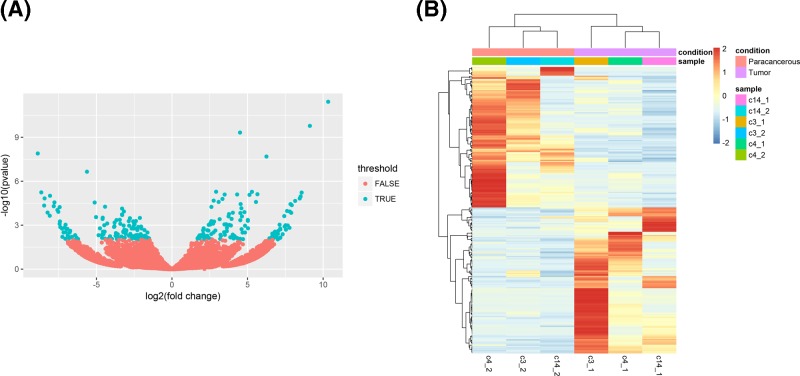
Differentially expressed circRNAs between cancer tissue and paracancerous tissue (**A**) The circRNA Volcano Plot. The horizontal axis represents the normalized difference (tumour group/normal group), the vertical axis is the normalized *P* value, and the blue and green dots in the figure are significant differentially expressed genes. (**B**) Double layer cluster heat map of differentially expressed circRNA, colour from blue to red represents circRNA expression from low to high. Each column in the map represents a sample, and each row represents a gene.

A total of 259 differentially expressed circRNAs in total were identified in the Volcano Plot based on a screening threshold |logFC| > 1, *P* value < 0.01, in which 132 were up-regulated and 127 were down-regulated (Figure 2A). Supplementary Table S3 lists the ten circRNAs with the largest difference in expression (up-regulation and down-regulation). Figure 2B is a heat map of the differential circRNA distribution.

### Using qRT-PCR to verify the accuracy of circRNA-seq data

To validate the differential expression of the candidate circRNA, qPCR experiments were used to verify the five maximally changed circRNAs of the 10 osteosarcoma and paracancerous tissue samples, mainly circ_32279, circ_24831, circ_2137, circ_6798 and circ_20403.

**Figure 3:**
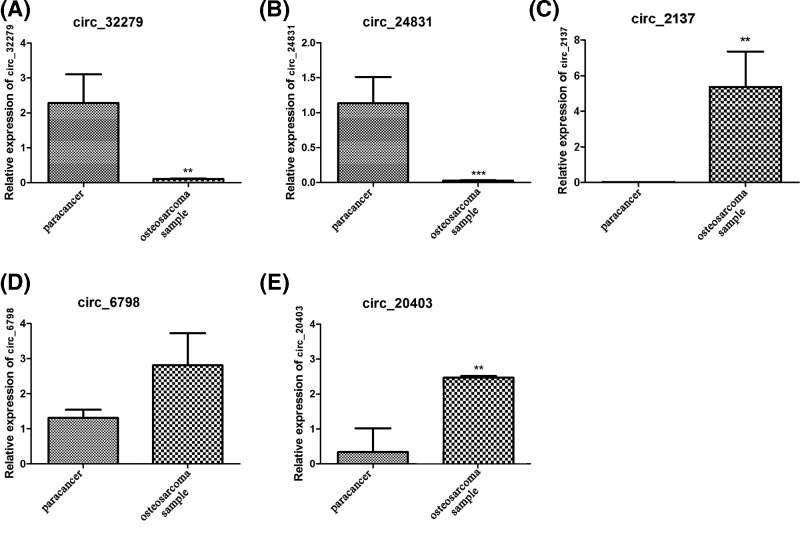
Relative expression of the differentially expressed circRNAs (**A**) circ_32279 gene; (**B**) circ_24831 gene; (**C**) circ_2137 gene; (**D**) circ_6798 gene; (**E**) circ_20403 gene. **P<0.01, ***P<0.001

Compared with adjacent tissues, circ_32279 and circ_24831 were significantly down-regulated in osteosarcoma tissues, while the expression level of circ_2137, circ_6798 and circ_20403 was higher than that in adjacent tissues, and the differences were significant between the expression levels of circ_2137 and circ_20403 but not significant with that of circ_6798 (Figure 3A–E).

### Pathway enrichment analysis of differentially expressed genes

GO function enrichment analysis and KEGG pathway enrichment analysis were performed on circRNA-hosting genes with significant differences. A total of 44 GO BP (biological process), 19 GO CC (cellular component), 15 GO MF (molecular function), 78 GO functions and 10 KEGG pathways were enriched.

**Figure 4:**
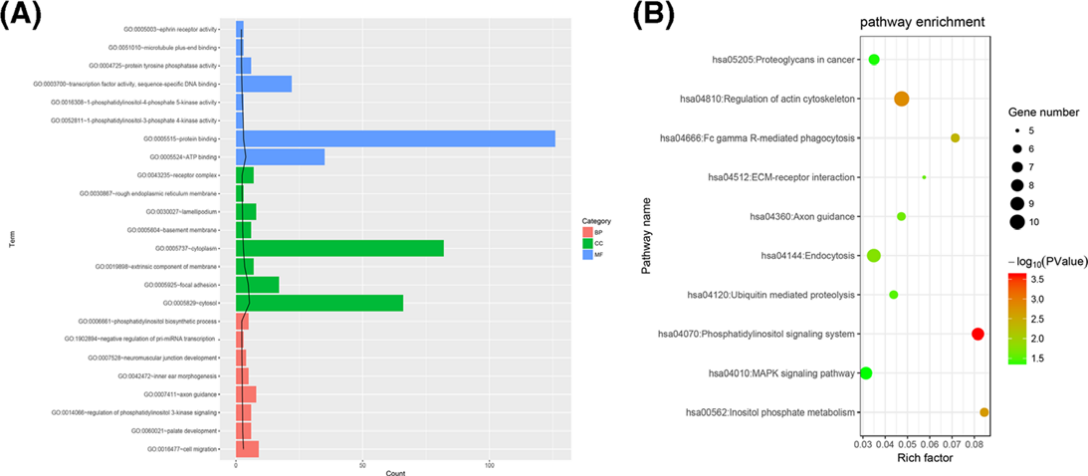
Pathway enrichment analysis of differentially expressed genes (**A**) GO function enrichment analysis results according to category. Term is the functional description information of GO and count is the number of different genes enriched to that term. The black trend line represents the −log10 value (*P* value). (**B**) KEGG pathway enrichment consequences. A value of −log10 (*P* value) is significant for enrichment. The greater the value is, the more meaningful the result. Gene number is equal to the number of genes enriched in the entry. Rich factor is the ratio of the number of genes in the pathway entry that is differentially expressed to the total number of genes in the pathway entry. The larger the rich factor, the higher the degree of enrichment. The ordinate is the name of the enrichment item.

**Figure 5:**
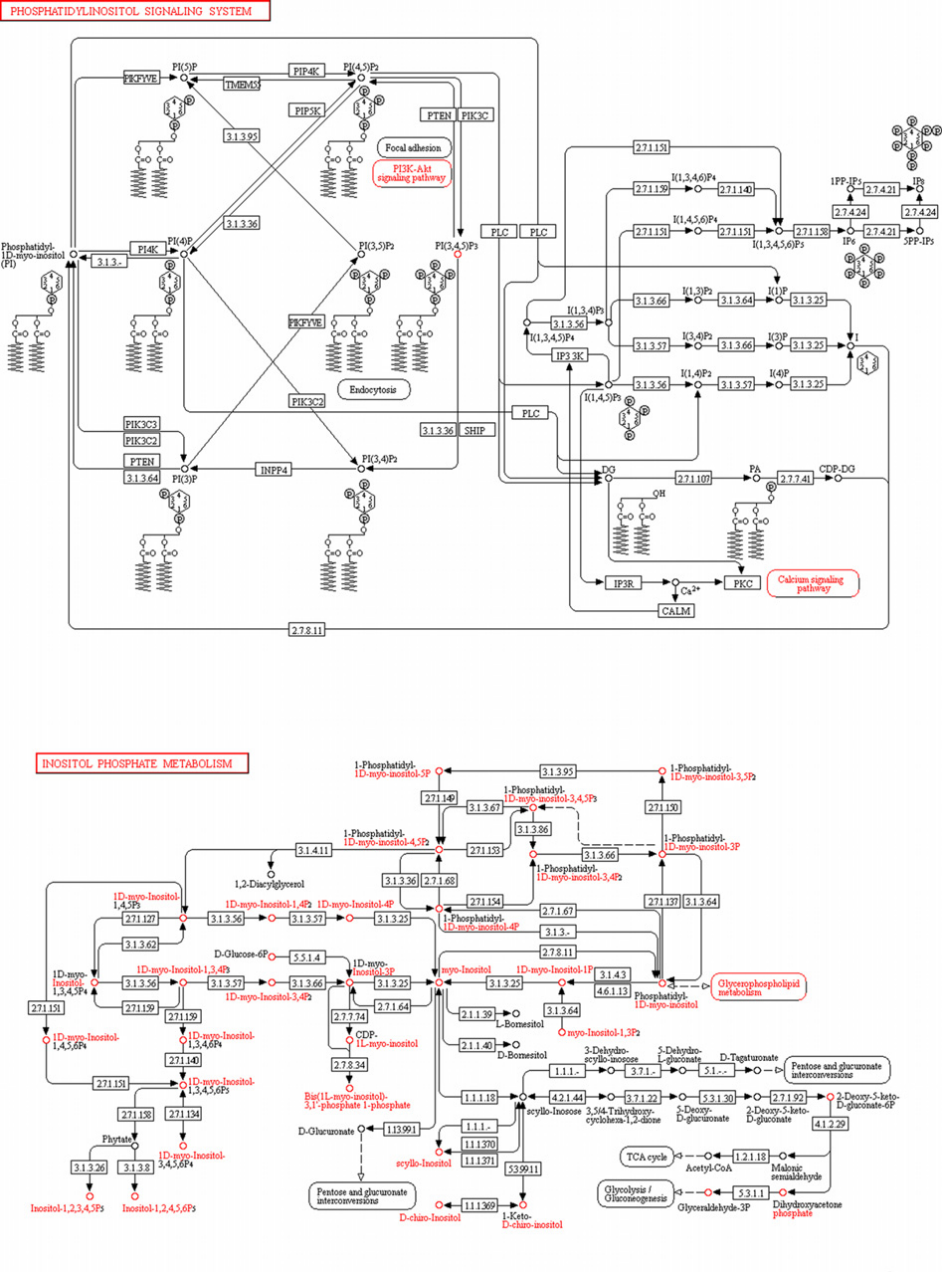
Genes are mapped to KEGG pathways by pathway analysis The signalling pathways of ‘phosphatidylinositol signalling system’ and ‘inositol phosphate metabolism’ indicate the modulation of tumour markers and are related to the outcomes of osteosarcoma.

The differentially expressed circRNAs were related to protein binding, ATP binding, gene expression in the cytoplasm, and cell migration (Figure 4A). The ‘phosphatidylinositol signalling system’ signal pathway and the ‘inositol phosphate metabolism’ signal pathway were the meaningful pathways (Figure 4B). The two pathways are presented in Figure 5.

### The networks of circRNA–miRNA identification

**Figure 6:**
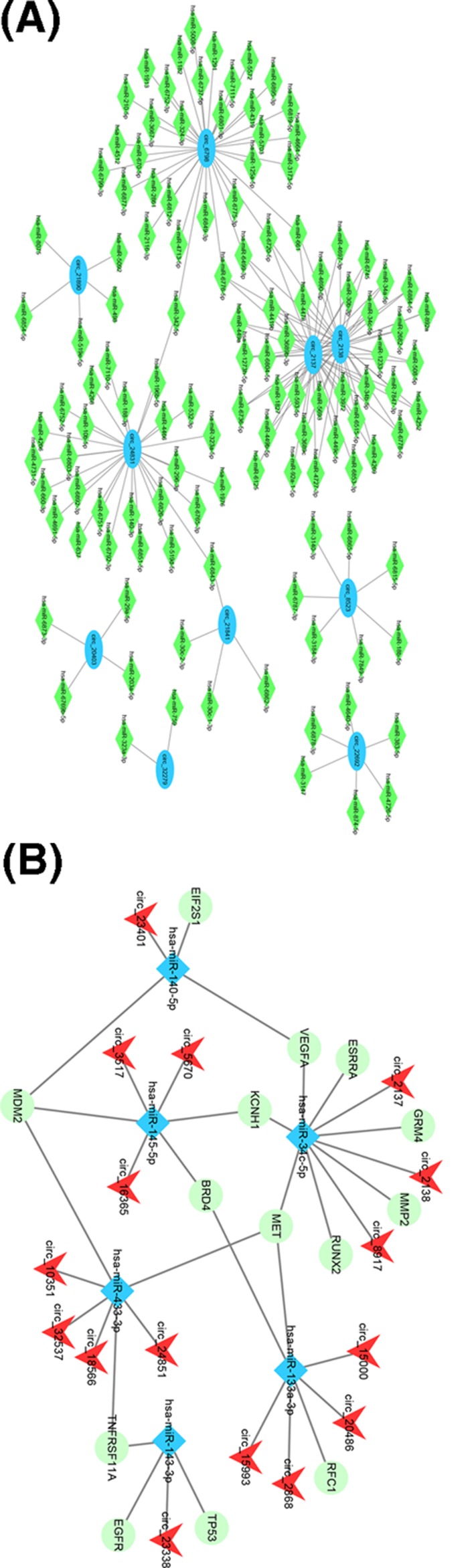
CircRNA and miRNA/gene network diagram (**A**) The miRNA–circRNA network diagram. The blue ellipse represents circRNA, the green diamond represents miRNA, and there is a link between the nodes to express the target relationship. (**B**) The circRNA–miRNA–gene network diagram. The red v-shaped nodes represent circRNA, the blue rhombic nodes represent miRNAs, the green circular nodes represent genes and straight lines indicate interactions between miRNA and circRNA or genes.

The analysis of bioinformatics shows that in mammalian cells, some circRNAs play a role in miRNA sponges. Some circRNAs (for instance CDR1as or miR-7) have been proven to have multiple target sites in order to bind with miRNAs to play a regulatory role in organisms. The miRNAs with the most targeted relationship with circRNA are presented in Supplementary Table S4. The miRNA–circRNA regulatory relationships are presented in Figure 6A.

### Construction of circRNA and disease-related miRNAs/gene network

From the analysis, 11 miRNAs and 44 genes associated with osteosarcoma were obtained. Six of the 11 miRNAs interacted with the differentially expressed circRNA in the frontal analysis. The miRWalk database was used to predict whether there was a regulatory relationship amongst them. The results shared by miRWalk, miRanda and TargetScan were selected as the final miRNA–gene relationship and included 6 miRNAs and 14 genes. Combined with the conventional analysis of miRNA–circRNA regulatory relationships, miRNA–circRNA was integrated into miRNA-associated miRNAs/genes, and a network diagram of circRNA and disease-related miRNAs/genes was obtained. Figure 6B shows a total of 16 circRNAs, whose information is shown in Supplementary Table S5. CircRNA can regulate gene expression by interacting with miRNA.

## Discussion

It has been confirmed that circRNA has an important function and plays a large role in the occurrence of illnesses. Additionally, circRNA has become a hot spot in RNA and transcriptome research. Owing to its unique structure and complex functional mechanism, the research on circRNA had a delayed start. CircRNAs have been shown to have a pivotal role in midbrain growth, Parkinson’s disease, Alzheimer’s disease and tumorigenesis. To elucidate the potential role of circRNA in the pathogenesis of osteosarcoma, we identified the expression markers of circRNA in the tumour tissues of patients with osteosarcoma and conducted functional analysis. In the present study, we observed 259 circRNAs that are differentially expressed within the neoplasm tissues of osteosarcoma, comprising of 132 up-regulated and 127 down-regulated circRNAs. From the Volcano Plot analysis, it was found that these differentially expressed circRNAs were similar to the down-regulated and up-regulated expression in the osteosarcoma patient group.

Cluster analysis showed significant differences in circRNA expression patterns between paracancerous tissues and osteosarcoma tissues. The results of the cluster heat map showed that circRNA expression in osteosarcoma was significantly up-regulated or down-regulated compared with that in paracancerous tissues. This suggests that there is a significant difference in the expression of some cyclic RNAs between osteosarcoma and paracancerous tissues.

In differentially expressed circRNAs, five circRNAs were selected to detect the relative expression in the two groups by real-time PCR. By comparison, we found that the difference in gene expression measured by the real-time PCR method and the high-throughput sequencing method was comparable, which confirmed the reliability of the results.

Liu et al. [10] found upregulated expression levels of Circ-NT5C2 in osteosarcoma tissues and cell lines. And gene chip technology was used to screen the expression profiles among genes of osteosarcoma and normal tissues [22]. In contrast, our study applied second-generation high-throughput sequencing technology. High-throughput sequencing is widely cited in the field of small molecule RNA or noncoding RNA (ncRNA) research. This sequencing method can easily solve the technical problems encountered in the detection of circular RNA by chip technology (i.e. short sequence, highly homologous). Moreover, the short sequence of cyclic RNA coincides with the length of high-throughput sequencing, thus not wasting the data. At the same time, the sequencing method can also find new circular RNA during the experiment. The disadvantage of a gene chip is that it is a ‘closed system’ that can only detect the features or limited variations of known sequences. The strength of deep sequencing is that it is an ‘open system’. Its ability to discover and find new information is inherently higher than that of gene chip technology.

KEGG pathway analysis and GO analysis showed that the differential expression of circRNA was involved in some biological processes and signal transduction pathways. Bioinformatics analysis of differential genes showed that the differential expression of circRNAs may be related to protein binding, ATP binding, gene expression in the cytoplasm, and cell migration. KEGG pathway analysis found that the ‘phosphatidylinositol signalling system’ signal pathway and the ‘inositol phosphate metabolism’ signal pathway may be related to the occurrence and development of osteosarcoma. Both the ‘phosphatidylinositol signalling system’ and ‘Inositol phosphate metabolism’ signal pathways are related to inositol pyrophosphate. The change in inositol pyrophosphate signalling might break the cellular energy homeostasis [23].

Increasing evidence suggests that circRNA plays a key role in numerous biological processes, one of which serves as a ceRNA or miRNA sponge and is competitive with MRE (microRNA response element, MRE) to combine the expression of miRNA-regulated genes. Several studies have reported that circRNA, upstream of the target gene, participates in the regulation of the miRNA, mRNA, and the ceRNA regulation mechanism of the circRNA–miRNA–gene, which affects the occurrence and development of a certain disease. For instance, circCDR1as a sponge for miR-7 regulates human epidermal growth factor receptors, which can contain more than 70 conserved binding sites of α-synuclein and insulin receptor substrates -2 and miR-7 [24]; in addition, cyclic Sry RNA acts as a sponge for miR-138 [25].

CircRNAs can be specifically bound to some protein factors, such as circRNA (circMbl) and splicing factor *muscleblind* (MBL / MBNL1) [26]. Circ-Foxo3 interacts with ID-1, E2F1, FAK and HIF1a and remains in the cytoplasm. It cannot play its anti-aging and anti-stress effects, which leads to an increase in cell senescence [27].

The cis-regulatory role of noncoding intronic transcripts on their parent coding genes [28] and back-splicing is regulated by common splicing factors and cis-elements, but unlike canonical splicing, the obtained circRNA can be translated to produce functional proteins [29]. There are some circRNA genes that regulate gene expression in the nucleus, such as EIciRNAs, which enhance the expression of its cis partner gene and emphasize the regulatory strategy of transcription control through the specific RNA–RNA interaction between UsnRNA and EIciRNA [30]. Some fairly stable circRNAs can be reverse transcribed and eventually inserted into the host genome as processed pseudogenes [31].

The translocation of established cancer-related chromosomes causes fusion-circRNAs, which are produced by the transcriptional exons of different genes affected by translocation. F-circRNAs facilitate cell transformation, promote cellular viability and therapeutic resistance, and have tumour-promoting characteristics *in vivo* models [32].

To date, limited studies have been conducted on the interaction between circRNA and miRNA in osteosarcoma. The circRNA–miRNA–gene network of osteosarcoma has been established based on the data obtained in the present study. The circRNA–miRNA–gene network can help make further efforts to understand the function of circRNA in the carcinogenesis of osteosarcoma.

In conclusion, the present study identified circRNA disorders in osteosarcoma. Bioinformatics analysis indicated that the disorder of circRNAs may be linked to the occurrence and development of osteosarcoma. This study can broaden the perspective of the gene study of osteosarcoma and lay a foundation for further studies on the role of circRNAs in osteosarcoma.

## Supporting information

**Supplemental Table S1:** Characteristics of the patients with osteosarcoma

**Supplemental Table S2:** Primers and DNA sequences used in this study

**Supplemental Table S3:** The ten circRNAs with the largest difference in expression (upregulation, downregulation)

**Supplemental Table S4:** The miRNA frequency

**Supplemental Table S5:** The circRNA information in the network diagram

## Abbreviations

ncRNA: noncoding RNA
RIN: RNA integrity number
