# Supplementary material for: Differential expression and bioinformatics analysis of circRNA in osteosarcoma
Source: Biosci Rep. 2019 May 17;39(5):BSR20181514. doi: 10.1042/BSR20181514 (PMC6522716; doi:10.1042/BSR20181514)
Supplement: Supplementary file 1 [file bsr20181514_Supp1.pdf]

Supplementary Tables

Table 1. Characteristics of the patients with osteosarcoma

Characteristics	
**Age, (year)**	
**Mean ± SD**	17.2 ± 5.6
**Range**	9-26
**Sex**	
**Male**	6 (46.1)
**Female**	7 (53.9)
**Ethnicity**	
**Han**	9 (69.2)
**Minority**	4 (30.8)
**Smoking status**	
**No**	12 (92.3)
**Yes**	1 (7.7)
**Drinking status**	
**No**	11 (84.6)
**Yes**	2 (15.4)
**Tumour location**	
**Femur, n (%)**	6 (46.1)
**Tibia, n (%)**	3 (23.1)
**Humeral bone, n (%)**	4 (30.8)
**Enneking stage**	
**I, n (%)**	4 (30.8)
**II, n (%)**	9 (69.2)
**Tumour size**	
**≤5 cm, n (%)**	4 (30.8)
**>5 cm, n (%)**	9 (69.2)
**Pathological subtype**	
**Osteoblastic type**	7 (53.8)
**Chondroblastic type**	3 (23.1)
**Fibroblastic type**	2 (15.4)
**Telangiectatic type**	1 (7.7)

Table 2. Primers and DNA sequences used in this study

Primer name	Primer Sequence (5′-3′)
**GAPDH-hF**	TGACAACTTTGGTATCGTGGAAGG
**GAPDH-hR**	AGGCAGGGATGATGTTCTGGAGAG
**circ_32279-hF**	TTGCAGTGTATGTTGTCGGGTA
**circ_32279-hR**	TGAGTCACAAGCAACATCTGA
**circ_24831-hF**	CCAGCCTGTCTACATCAA
**circ_24831-hR**	TAAATCCGTGCTTCTCC
**circ_2137-hF**	TAGACTAATAAGTGACAGGGTT
**circ_2137-hR**	TGTTGGAAGTGGAGGGT
**circ_6798-hF**	CAGGGACGGCTTGATGG
**circ_6798-hR**	GCGAAGACAAAGTCGAGGG
**circ_20403-hF**	CTTTACCCAGAAATACCCAC
**circ_20403-hR**	TTTCCTGGATAGACCTGTTG

Table 3. The ten circRNAs with the largest difference in expression (upregulation, downregulation)

circ_name	location	geneName	logFC	logCPM	F	P value	FDR
**circ_32279**	chr7:32672154-32678977:-	DPY19L1P1	-8.8794	7.865574	32.37816	1.28E-08	5.88E-05
**circ_24831**	chr6:149208121-149285699:+	UST	-8.64624	6.850988	20.56511	5.78E-06	0.010905
**circ_21841**	chr12:50824277-50835426:+	LARP4	-8.45639	6.593937	16.61752	4.58E-05	0.029042
**circ_21890**	chr12:66603235-66611015:+	IRAK3	-8.42778	6.744892	18.7573	1.49E-05	0.015392
**circ_22692**	chr17:48814320-48819092:+	LUC7L3	-8.23093	6.407996	14.53618	0.000138	0.059889
**circ_8523**	chr14:71880664-71996087:+	SIPA1L1	8.451599	6.765993	18.73325	1.51E-05	0.015392
**circ_2138**	chr13:64576517-64608670:-	AK057471	8.512857	6.795144	19.34833	1.09E-05	0.012547
**circ_2137**	chr13:64564228-64608670:-	AK057471	8.579295	6.934739	20.5156	5.93E-06	0.010905
**circ_6798**	chr16:29917108-29917448:+	ASPHD1	9.122766	8.217934	40.84021	1.67E-10	1.54E-06
**circ_20403**	chr2:106774513-106782539:-	UXS1	10.33314	8.42944	48.26512	3.79E-12	6.96E-08

Table 4. The miRNA frequency

miRNA	Frequency
**hsa-miR-4691-5p**	24
**hsa-miR-4731-5p**	23
**hsa-miR-3692-5p**	22
**hsa-miR-6764-5p**	22
**hsa-miR-8089**	22
**hsa-miR-6851-5p**	21
**hsa-miR-4685-5p**	20
**hsa-miR-762**	20
**hsa-miR-3064-5p**	19
**hsa-miR-5193**	19

Table 5. The circRNA information in the network diagram

circ_name	location	Host_geneName	isoformName	Regulate
**circ_2137**	chr13:64564228-64608670:-	AK057471	uc001vii.1	UP
**circ_23401**	chr18:6263945-6312055:-	L3MBTL4	ENST00000400105	DOWN
**circ_2138**	chr13:64576517-64608670:-	AK057471	uc001vii.1	UP
**circ_20486**	chr2:122260742-122287901:-	CLASP1	uc010yyy.2	UP
**circ_2868**	chr12:70193988-70195501:+	RAB3IP	ENST00000247833	DOWN
**circ_8917**	chr19:2213537-2214595:+	DOT1L	ENST00000398665	UP
**circ_18566**	chr4:183245098-183268082:+	TENM3	ENST00000513201	UP
**circ_32537**	chr1:17668437-17668897:+	PADI4	uc009vpc.2	DOWN
**circ_24851**	chr6:154520801-154535410:-	IPCEF1	NM_001130699	DOWN
**circ_16365**	chr6:145148746-145157074:+	UTRN	NM_007124	DOWN
**circ_3517**	chr11:6962801-6977013:+	ZNF215	uc001mez.1	DOWN
**circ_10351**	chr7:23381682-23391205:-	IGF2BP3	NM_006547	UP
**circ_15993**	chr6:47251673-47254331:-	TNFRSF21	NM_014452	UP
**circ_5670**	chr17:34881058-34884033:-	MYO19	ENST00000431794	UP
**circ_23338**	chr19:8619360-8619627:-	MYO1F	NM_012335	DOWN
**circ_15000**	chr9:88233897-88248289:-	AGTPBP1	uc004aod.4	DOWN
